# Prevention in Autism Spectrum Disorder: A Lifelong Focused Approach

**DOI:** 10.3390/brainsci11020151

**Published:** 2021-01-24

**Authors:** Konstantinos Francis, Georgios Karantanos, Abdullah Al-Ozairi, Sulaiman AlKhadhari

**Affiliations:** 1 Kuwait Centre for Mental Health, Kuwait City 70031, Kuwait; 2 Hellenic Scientific Network for ASD, 15231 Athens, Greece; karant-g@otenet.gr; 3 Department of Psychiatry, Faculty of Medicine, Kuwait University, Kuwait City 46300, Kuwait; alozairi@gmail.com (A.A.-O.); alkhadhari@hsc.edu.kw (S.A.)

**Keywords:** Autism Spectrum Disorder, prevention, life course approach, risk factors, early intervention

## Abstract

Autism Spectrum Disorder (ASD) is a complex highly heritable disorder, in which multiple environmental factors interact with the genes to increase its risk and lead to variable clinical presentations and outcomes. Furthermore, the inherent fundamental deficits of ASD in social attention and interaction critically diverge children from the typical pathways of learning, “creating” what we perceive as autism syndrome during the first three years of life. Later in life, training and education, the presence and management of comorbidities, as well as social and vocational support throughout the lifespan, will define the quality of life and the adaptation of an individual with ASD. Given the overall burden of ASD, prevention strategies seem like a cost-effective endeavour that we have to explore. In this paper, we take a life course approach to prevention. We will review the possibilities of the management of risk factors from preconception until the perinatal period, that of early intervention in the first three years of life and that of effective training and support from childhood until adulthood.

## 1. Introduction

Autism Spectrum Disorder (ASD) is a neurodevelopmental condition characterised by deficits in social communication; social interactions and restricted, repetitive patterns of behaviours, interests or activities [1]. ASD is a highly heterogeneous condition with the concept of a spectrum capturing the differences among autistic people in terms of symptoms; intelligence and language abilities; neuropsychological underpinnings (cognitive style, information processing and sensory deviations); aetiology and comorbidities, as well as the levels of functioning, adaptation and wellbeing.

In 2010, the global burden for ASD was calculated to be 111 Disability-Adjusted Life Years per 100,000 persons, with a very conservative estimated ASD prevalence of 0.76 [2]. Note that, according to the CDC latest (2020) report [3], the prevalence estimate for ASD has soared up to 1.85%. However, this still has the highest burden among mental disorders with an onset in childhood, higher than that of Attention Deficit Hyperactivity Disorder (ADHD) and conduct disorder combined, and the fifth among mental disorders after major depressive disorder, anxiety disorders, schizophrenia and bipolar disorder [4]. The lifelong financial costs per person with ASD in the USA were calculated in 2014 to be $2.4 million if the person had comorbid intellectual disability and $1.4 million if he/she did not, with a total cost that surpassed the entire Gross Domestic Product of 139 countries around the world [5]. These figures, along with the fact that no medication or other biological therapy for ASD is currently available (albeit recent advances) [6], render the implementation of prevention strategies a logical, cost-effective and rather imperative step for the scientific community, the health authorities and ASD advocates.

### 1.1. Development of ASD

Before we discuss prevention strategies, we have to conceptualise the way autism is formed and evolves over the lifetime of each person. This will help us to determine the ways and timing of our preventive interventions. Family and twin studies offer strong evidence for a predominant genetic aetiology in ASD, with over 100 susceptibility genes identified to be strongly linked to autism [7,8] and heritability estimates ranging from 50–95% [9]. Albeit the preponderance of genetic factors, the literature suggests that ASD could result also from effects deriving from environmental risk factors (thus, the high but not full concordance in monozygotic twins), as well as from gene x environment interactions [8,10]. However, a deeper understanding of epigenetics has underscored the conceptualisation of disorders as a “mismatch between individual and a specific environment, rather than an abnormality per se” and can explain autism as an expected reaction to a nonoptimal environment for the individual in a physical and psychosocial context [11]. Specifically, in ASD, it is hypothesised that genetic and environmental factors lead to a subject’s atypical patterns of interactions with the environment, with impaired engagement in social interactions. These “risk processes” will result, on the one hand, in abnormal social and linguistic brain circuitry and, eventually, mediate the development of full-blown ASD (i.e., “autism will create itself”). On the other hand, they may intervene through epigenetics in the expression of the susceptibility genes, further strengthening their effects, as if in a vicious cycle [12]. Finally, as one would expect, the overall outcome of the disease and its burden is further determined by social, psychological and biological factors from childhood to adolescence and to adulthood through constant feedback loops between the environment and the indices of the disorder.

### 1.2. Prevention Approaches

Commonly, prevention in mental health can be distinguished in three forms. First, as a primary prevention that aims to reduce the incidence of a disorder and targets broad populations. Next, as a secondary prevention targeting selected at-risk groups aiming to reduce the prevalence of the disorder (or its severity). Thirdly, as a tertiary prevention for indicated subjects with the aim to preserve functional adaptations and the wellbeing of the person, as well as to avoid relapses [13]. These strategies should be applied in a life course approach, from the prenatal period to old age, with a variable focus for each type depending on the age of the targeted subjects. Due to the conceptual overlap between the types of prevention and the population they target (e.g., a primary intervention could target an at-risk population only), as well as that of secondary and tertiary preventions with actual treatment interventions, a more productive framework could be to dissect prevention in mental health based on the time the relevant measures are taken. 

In this framework, and based on the development of autism described above, primary prevention for autism would mean efforts, preconceptionally and during pregnancy, to manipulate its root causes [14] in an attempt to increase resilience and reduce occurrence [15]. These targets are the developmental risk and preventive factors known to participate in the etiopathology of ASD in the interplay with genetic causes. During the first two-to-three years of life, prevention measures (secondary or tertiary) will be aimed at altering the developmental cascade that started before birth [16] and, thus, alleviating or even preventing the emergence of autistic symptomatology [12]. From childhood and adolescence until adulthood and old age, and with the ASD fully present, prevention strategies are mainly of the secondary and tertiary levels in an attempt to increase or preserve the level of adaptation and the wellbeing of the autistic person. Furthermore, they will prevent the appearance of secondary problems, such as extreme disruptive behaviours, depression, transition difficulties, etc. In a broad sense, all treatment interventions for these ages display an inherit preventive value. 

In this paper, we will review the possibilities, as well as the research data available, for prevention efforts during these three periods of the life of an autistic person.

## 2. Prevention Strategies

### 2.1. Preconception/Perinatal Period

As presented above, the root causation of ASD is multifactorial, with multiple genes interacting with each other and with environmental factors, both prenatally and, most probably, early postnatally, as the phenomenon of developmental regression demonstrates [12]. Genes represent the baseline susceptibility, but epigenetic phenomena deriving from a “toxic environmental load” and acting during the right critical time window [17] result in physiological changes that overcome the individual’s resilience and adaptation, finally translating into a neuro-atypical phenotype [18]. In this context, prevention seeks the optimal manipulation of these factors that can render the causal constellation insufficient to produce the ASD phenotype or at least the full-blown one [14]. 

The factors that are backed by more scientific support or are the focus of discussion and research can be divided into three categories: those we cannot manipulate for various reasons, those that represent a matter of general community health and those that are more specific for the development of ASD. The latter need to be addressed mainly, but not exclusively, in high-risk populations, e.g., with a positive family history of ASD.

In the first category, the main example is the parental ages (older fathers, older or very young mothers and increased age differences between parents) that increase the risk of ASD [19]. Similarly, assisted reproductive technology (ART) is often a mandatory choice to address a couple’s infertility. Albeit concerns from clinicians, the role of ART in the risk of ASD is not yet very clear, especially when ART is examined as a whole [9]. Specific relevant treatments (e.g., intracytoplasmic sperm injection), though, may have a greater additive risk for ASD [20]. The assessment of the possible risk for ART becomes more pertinent if the techniques are used for gender selection, given the substantially higher incidence of ASD in boys (4:1) and the higher recurrence risk for boys in the presence of a sibling already diagnosed as ASD [21]. The overall reduction in risk by gender selection (choosing a female) is calculated as 3% if the couple has one autistic child and 15% if they have two autistic children [22]. The residual risk for ASD from gender selection could be increased to the extent (unknown) that ART itself increases the risk of ASD, whilst an increase in birth defects and other adverse pregnancy outcomes related to ART should also be factored into the decision. Ethical issues are also pertinent to this “preventive” approach [22]. Overall, the choice of gender selection seems more rational when the reduction in risk will be substantial, as in the case where there are already two autistic siblings, and it is less attractive when the reduction in risk is less, e.g., when there is just a positive family history of ASD.

The factors of the second category represent prevention targets for the general population, as their effects extend beyond the ASD pathoaetiology, and prevention measures should be addressed for the whole population. According to the “prevention paradox”, the expected benefits on the incidence of ASD of such effective large-scale prevention measures can outperform the ones from measures aiming only at the high-risk subpopulation [23].

Congenital hypothyroidism is implicated in mental disabilities and maybe in the pathophysiology of ASD [24], but it was recently shown that maternal hypothyroidism during pregnancy also increases the risk of ASD [25,26]. Testing for congenital hypothyroidism is a well-established practice in most countries that should be coupled with relevant testing during pregnancy. 

Air and chemical pollutions are also a matter of general public health. Data for the effect on ASD risk due to air pollution (i.e., hazardous air pollutants and air pollutants criteria as defined in the USA) and endocrine-disrupting chemicals (like those found in pesticides, plastics, fragrances, etc.) are inconclusive [9,14]. However, studies with more sophisticated designs for the measurements of actual exposure have implicated some pesticides [14]. Interestingly, the effects of prenatal pesticide exposure can be mitigated by a higher folic acid intake in the first month of pregnancy [27]. Data for ASD and other health effects render imperative a tighter control by the relevant environmental protection agencies on the allowed “safe levels” of various pollutants and on the use of pesticides and other chemicals. 

Maternal smoking during pregnancy is correlated with risks for pregnancy and birth complications (miscarriages, decreased growth, complications during labour, preterm birth, stillbirth and sudden, unexpected death in infancy) [28], as well as long-term effects like asthma and behavioural problems or ADHD [29]. Several meta-analyses reported a noncontribution to ASD risk [30], but in more sophisticated analyses of confounding factors like second-hand smoking and the severity of smoking, a positive correlation is revealed [30,31]. Smoking cessation programs have to be implemented during pregnancy and particularly in women from a lower socioeconomic status.

Apart from the multiple consequences of reduced or increased foetal growth and preterm deliveries on the general health of the child, a higher prevalence of ASD has been systematically reported in children born prematurely [32], as well as in those with bilateral deviances in foetal growth [33]. It is not clear if these are risk factors per se or the expression of other factors, but general measures for the optimal clinical care of pregnant women should be applied to reduce their incidence [34]. Relevant to these, studies consistently report a higher prevalence of pregnancy complications and obstetric suboptimality in children with ASD [35,36], including preeclampsia [37]. However, it seems more possible that this could be rather an epiphenomenon of the ASD or the result of a shared risk factor [38]. On the contrary, maternal obesity and gestational diabetes, along with other metabolic conditions (elevated glucose, triglycerides, cholesterol, leptin and proinflammatory immune markers), may substantially increase the risk for ASD [39,40] through multiple pathways [14]. Both for ASD risk reduction and for the prevention of short- and long-term health risks for the mother, developing foetus and offspring, relevant follow-up and treatment programs should also be applied [41].

The risk factors of the third category are more pertinent to ASD, without, of course, lacking more general effects on the offspring. These factors should be the focus of the prevention in high-risk cases. Immune reactions during pregnancy represent additive risk factors, whether these were the results of infections [42]—with congenital rubella being the historical paradigm [43]—or maternal autoimmune conditions [44]. Maternal influenza, other viral infections in the first trimester and bacterial infections, as well as prolonged fever in the second trimester, were found to increase the risk of ASD up to three-fold [45,46]. These latter data could explain the seasonality of the births reported in several studies [47], along with vitamin D levels or pesticide use [48]. Towards mitigating the risk of ASD, general preventive measures to avoid infections should be implemented on a whole population level. For mothers at high risk for ASD, vaccination programs should be offered, especially for such well-established risk infectious factors like rubella [49], while a more aggressive treatment of the fever itself should be the clinical practice. In the case of the circulation of maternal autoantibodies, several therapeutic interventions have been proposed as possible preventive measures [44]. 

Short interpregnancy intervals (IPI) (<12 months) increase the risk of autism, possibly through nutrient deprivation [9,14] (which can be further enhanced if the mother breastfeeds) and the subsequent negative effects on birth weight and risk of small-for-gestational-age. The latter two effects have been shown to be mitigated by folic acid use [50]. An alternative explanation for this finding could be bias from cultural parameters and/or lifestyle ones, since a long IPI (>60–84 months) has also been associated with a higher ASD incidence [9,14]. Thus, in high-risk families, short IPIs should be avoided, and in the case of one, intense supplementation with vitamins, folic acid and minerals, as well as generous nutrition intake during the new pregnancy, could play a protective role. 

Although maternal folic acid supplementation is a common preventive measure proposed by many national authorities like the CDC in USA [51] to prevent neural tube defects [52], its connection to ASD is less conclusive in the literature [53]. However, some studies reported significant protective effects when taken around conception [54] and, especially, in the presence of inherent inefficient folate metabolism [55]. Preconception supplementation in planned pregnancies in high-risk families is a safe and promising prevention measure to help decrease not only the ASD risk but, also, other behavioural problems [56] and delays in language [57].

Other nutrient deficiencies like vitamin D, iron and polyunsaturated fatty acid (PUFA), especially omega-3, have also less robustly been correlated with a higher risk for ASD [9]. Iron is a critical mineral for brain development and functioning, but the data from two studies for the specific risk for ASD are contradictory [14]. Omega-3 supplementation or fish consumption is associated with a higher IQ and better neurodevelopment, but its correlation with ASD is less clear [14]. However, a big prospective study showed that a higher fat fish consumption prenatally had a protective effect on ASD, even after controlling for the mercury levels bioaccumulating in them [58]. The role of vitamin D in brain development and its functions has been demonstrated in multiple animal and human studies, but its specific role in ASD is still unclear, given the multiple confounding factors in the assessment of their relationship [59]. However, a small open-label prospective study in siblings of probands with ASD found that vitamin D supplementation during pregnancy and breastfeeding or for the first three years of life for those infants that did not breastfeed led to a four-fold reduction in the recurrence rate [60], possibly through the enhancement of insulin-like growth factor 1 (IGF-1) levels (see below) [61]. Vitamin D alone or with omega-3 supplementation has been also tried as a secondary measure of prevention in children with ASD, with equivocal results [59,62]. Given the current state of data and the relative safety of such an approach, relevant supplementation during pregnancy could be suggested, at least for omega-3 and vitamin D. 

Lately ASD has been hypothesised to be an insulin-like growth factor 1 (IGF-1)-related brain dysconnectivity; thus, measures to increase the availability of this molecule postnatally could represent a plausible prevention [61]. This can be attained through breastfeeding for up to one year, and research data has shown that children with ASD were significantly less likely to have been breastfed [63]. Of course, an adequate breast milk IGF-1 concentration is required, a figure that we currently have not determined. As mentioned above, prenatal or postnatal vitamin D supplementation also increases the circulating IGF-1, offering significant protection [60,61]. Finally, the administration per os of modified forms of IGF in the first year of life can increase the availability of free IGF-1 and protect the hypomyelination of the brain that leads to its dysconnectivity. Although extended breastfeeding could be easily applied as a general prevention measure, intensive vitamin D supplementation or the use of IGF forms can only be suggested for high-risk infants (e.g., siblings of ASD probands, preterm infants, etc.) and, especially, to those with low levels of IGF-1 in the umbilical cord blood at delivery. The effectiveness of these interventions, however, awaits confirmation from relevant studies.

Several medications administered during pregnancy have been incriminated as ASD risk factors. These include the serotonin-reuptake inhibitors—SSRIs (although some debate still exists around the effects of depression itself) [64,65], the antiepileptics, especially valproate [66] and anti-asthmatic β−2 adrenergic receptor agonists [67]. The avoidance or the substitution of these medications, when possible, sounds like a logical preventive measure, especially for high-risk populations.

The use of modern high thermal intensity ultrasound in the first trimester of pregnancy of children with ASD was found to correlate with more repetitive behaviours and lower nonverbal IQ, especially so in boys with genetic (ASD-associated Copy Number Variants/CNVs) vulnerabilities [68]. The effect is plausible considering the effects of an ultrasound in utero on the brain structure and behaviours in animal models. Since the toxic window covers the first trimester, avoidance or reasoned use of an ultrasound during this period may decrease the overall risk for ASD.

Finally, albeit the widespread public worry about the impact of vaccinations and, especially, of the Measles Mumps Rubella vaccine, research has proven that there is no correlation between them and the risk of ASD [69]. The modern tendency, though, of parents avoiding the vaccination program of their children not only will have no effect on the ASD risk but also has detrimental effects, such as a substantial increase in morbidity and mortality from preventable diseases, as stated by the WHO, the CDC and the health agencies of many countries. 

### 2.2. Early Intervention in the First Three Years of Life

Based on the social motivation theory, most of the characteristics for ASD impairments in social communication are the “adaptive” consequences of the infant’s inherent inability to pay attention and integrate important social stimuli [12]. This heritable primary impairment [70] characterises the above-mentioned “mismatch” with the demands of the environment, leading the infant to adopt aberrant compensatory reactions and to relevant impairments, e.g., a failure to develop expertise in faces or failure to develop language. This hypothesis opened up the possibility of an intervention through enriching the environment and enhancing parent-child interaction in an attempt to guide behavioural and brain development towards more normal pathways. The plausibility of such an endeavour is supported by animal models where the effects of genetic and environmental hits are mitigated by environmental enrichment [12]. 

However, one of the major critical points of this approach (other critical points are the extent of the targeted impairments and that of the second domain i.e. range of interest/flexibility, as well as the presence of comorbid intellectual disability and/or language impairment) is that this intervention should be implemented as early as possible to increase the chance and the amplitude of the developmental redirection. During roughly the first two years of life, brain development is particularly sensitive to experiences but progressing maturation diminishes the brain plasticity [71]. This underscores the importance of a real early identification of the infants at risk, and research has already produced substantial advances on the issue. It was shown that differentiation from typical developing children becomes more evident by the age of 12 months and even more at 18 months with relevant clinical instruments [12]. Neurobiological markers such as event-related potentials to faces or to speech sounds and the rapid growth of the brain from birth to 12 months, especially if followed by a deceleration after 12 months [72,73], are being explored. In a series of recent studies, Bosi et al. used an EEG to accurately detect later diagnosed ASD in infants as early as three months of age, a promising marker if larger studies prove its clinical applicability [74]. Developmental regression can also be used for a reliable early identification of cases and paediatricians and family doctors should be sensitised to screen for it, as parents may not volunteer the information, especially in more subtle cases. A recent study showed that while, retrospectively, parents report regression in 30–40% of the cases, in prospective baby sibling studies, the prevalence of regression was doubled [75]. Clinical “red flags” (see the lists by the CDC or AutismSpeaks), and, especially, parental concerns [76] can also lead to early identification. The presence of behaviours compatible with ASD or of general developmental deviances should alert the clinician for a detailed assessment and the commencement of intervention even with a probable and unconfirmed diagnosis, as, in these cases, sensitivity is more important than specificity. A wait and see approach with the presence of parental concerns or red flags can have a detrimental effect in the long-term adaptation outcomes of the child. 

To demonstrate the plausibility of such prevention strategies, we opted to look at the actual long-term results of early interventions. Although there are several well-described interventions with adequately supportive published data, such as JASPER [77], we will present herein data from two only early interventions: 

Paediatric Autism Communication Therapy (PACT) [78] aims to improve the social communication and patterns of restrictive and repetitive behaviours through parent–child interaction and play. Through video feedback, parents learn to create opportunities for communication and adapt interactions in their day-to-day lives. The therapists encourage and empower parents towards adapted communication tailored to their child’s individual competencies in order to attain the highest possible level of interaction. The therapy consists of twelve 1.5-h fortnightly sessions over six months, with optional monthly maintenance sessions for a further six months. Between sessions, parents are asked to practice PACT strategies for at least 30 min daily during play or natural interactions with their child so PACT is gradually introduced into their everyday family life.

In the initial assessment of the intervention over treatment as usual, equivocal results were reported, with no significant effects on the prespecified primary outcome measures—total score of the Autism Diagnostic Observation Scale – Generic (ASOS-G)—a fact that could be attributed to the lack of its sensitivity to change (for this reason, the Brief Observation of Social Communication Change (BOSCC) was developed from the ADOS) [79]. A further issue could have been that the mean age of the study sample was 45 months (younger group 24–42 months), a point pertinent to decreasing plasticity mentioned above and also reported to have a significant long-term effect in early intervention studies [80]. However, when the results were reanalysed with the newer ADOS-2 calibrated severity scores (CSS), a significant difference was observed for the PACT intervention [81]. Furthermore, positive results were reported for parent-rated outcomes (language and social communication), as well as for assessor-rated ones (parental synchronous response to child, child initiations with parent and for parent–child shared attention). These were considered positive predictors for later social and communication functioning [82]. 

Since the gains in dyadic interaction were found to mediate over 70% of the communication improvements in the dyad and, also, of the symptom changes in ADOS [83], it was hypothesised that, through attaining this proximal target of the intervention, enhanced adaptive gains would be detected at a longer follow-up. Indeed, after more than five years, the PACT group showed significantly lower ADOS CSS, with gains in both social communication and (interestingly) in repetitive behaviour symptoms [81]. A further interesting finding was that, although the initial gains in parent synchrony disappeared in the follow-up, meaning parents lost the skills learned initially, those in child initiations were preserved, proving the long-lasting effects of the intervention. 

The Early Start Denver Model (ESDM) [84] is a comprehensive, developmental, relationship-focused and play-based approach aiming at helping autistic children develop interest in others, social communication skills, play skills, relationships and language. The ESDM also builds on the Applied Behaviour Analysis (ABA) strategies, using a child-tailored program with objectives, goals and activities targeting skill development. The therapist also teaches the parents and other caregivers how to implement the program whenever they are with their children. The child’s progress is reviewed regularly every three months to update this plan.

In the original randomised, controlled trial study [85], toddlers aged below 30 months received a mean of 15.2 h/week of ESDM intervention, and parents were trained so as to deliver at least five h/week (actual mean 16.3) of ESDM for two years. They also received 5.2 h/week of other therapies. The controls received the usual community interventions—COM (mean 9.1 h/week of individual therapy and 9.3 h/week of group interventions) available in their area. No major significant differences were observed in the first year of treatment, while significant ones were attained in the second year in the cognitive domains of language and adaptive functioning but not on the severity of the core autism symptoms as measured by the ADOS. Probably, the most significant finding was that the ESDM group’s adaptive functioning in everyday life, contrary to what is usually observed in ASD, as well to what happened in the COM group, maintained the same pace of development as the neurotypical children, without falling further behind. The results were only partially replicated in a larger multicentre trial [86]. It is noteworthy, though, that ESDM intervention was found to normalise atypical patterns of EEG activity related to social attention and engagement, probably underlying the improvements in social behaviours [87]. Apart from this highly intensive one-on-one intervention, ESDM has also been tried as parent coaching format [88] and in group-based community settings [89], with encouraging results.

In the follow-up study after two years [90], children in the ESDM group retained the gains of the original study, and the COM group caught up with them in all domains except in the adaptive scores (composite and socialisation) and the reduction in ASD symptom severity (ADOS total and repetitive behaviours). It is noteworthy that this effect was achieved with the ESDM group receiving fewer hours/week of services (and ABA ones) compared to the COM group, and these services were more in group setting, which supports the cost-effectiveness of this intervention [91]. 

Taking the overall data from these two different early interventions, one could argue that a secondary prevention during this period of life is not only feasible (albeit the shortcomings of the available interventions) but, also, a cost-effective and mandatory endeavour that can even reduce the prevalence of the disorder. This is especially true on an individual basis, where such interventions can help mitigate the long-acting effects of the inherit impairments, redirecting the person towards a more typical developmental trajectory. 

Basic blocks of these early interventions are the increase in the child’s social interest in interactions, the enrichment of his/her responses, the synchronisation with others and learning in naturalistic settings. Towards this end, there are several components from different programs that can be combined, modified and adapted on a personalised basis. These should be addressed to all infants showing probable ASD behaviours from as early as 12 months and, especially, to those at risk, such as the siblings of autistic probands, those born prematurely and those with severe visual or hearing difficulties.

### 2.3. From Childhood to Aduldhood

While, in the previous sections, we focused on prevention strategies aiming mainly at reducing the incidence, prevalence and severity of autism symptoms, in this third part, we discuss preventive measures to maximise and sustain adaptation. Adaptive functioning, although not completely independent either from autism (symptom) severity or the IQ [92], is not determined exclusively by them but, rather, captures the transaction between an individual and his/her environmental contexts [93]. This is reflected in the severity classes in the DSM-5 that are based on the level of needed support. It is this support throughout the lifespan that can alter the rather negative outcomes of autistic people in adulthood [94]. Thus, any treatment, measure or support offered from the preschool age to old age has a preventive component for the individual’s overall quality of life.

There are some advantages in the (unusual in the literature) conceptualisation of interventions for autism after the first years of life, also, as a preventive strategy. From the individual’s perspective, the therapeutic/supportive measures not only ameliorate the current quality of life but, also, prevent multiple detrimental future effects that will be the expected consequences of the current “difficulties” the specific measures try to address. For instance, sensory integration therapy/techniques not only positively affect behaviour, communication and learning here and now but will also protect the person from the negative cascade seen if these are left unattended, e.g., hyper- or hypoactivity, reduced play and learning, challenging behaviours, etc. [95]. From the family and immediate environment agents’ (school, employers, etc.) perspectives, viewing the efforts put into various interventions (e.g., implementing a visual schedule at home, classroom adaptations or allocating support for an ASD employee) as an effective way to attain a major future effect (e.g., to avoid disruptive challenging behaviours) will encourage and enhance the determination of these agents to implement the intervention. Finally, from a health system perspective, the justification and cost-effectiveness of any intervention considered for implementation relies heavily on its prevention gains (e.g., see the relevant ESDM study [91]).

A further notion for the preventive effects of the interventions after the first critical years of life is that skill gains are not necessarily acquired through changes in the fundamental underlying deficits and a return to more normal trajectories but, rather, through alternative compensation mechanisms, probably similar to what we see in dyslexia [96]. This different path is often reflected in acquired skills that, though they enhance adaptation, can be rather inflexible, not generalised, bound to circumstances and not always fully functioning [93].

#### 2.3.1. Preschool to Prepubertal Period

The majority of interventions are studied and applied in preschoolers and younger children. A detailed individual plan should be the basis of all the chosen components of the intervention, depending on the strengths and weaknesses of the person and with clear short- and long-term goals. In principle, these extremely diverse intervention efforts have the following common preventive goals:

(1) Protect future autonomy at the highest possible level through increasing communication and social skills, as well as daily living skills. Continuation of the interventions from the previous stage, intensive behavioural interventions and, especially, those with developmental components like the Naturalistic Developmental Behavioural Intervention (NDBI) or the social communications/emotional regulation/transactional support (SCERTS), the TEACCH program (Treatment and Education of Autistic and related Communication handicapped Children) and a plethora of other targeted interventions (social skills, speech and language and occupational therapies) claim to target increased adaptation [93,97].

(2) Avoid the emergence of challenging behaviours. [98] This could be the result of interventions assuring that communication abilities meet the respective needs (e.g., use of Picture Exchange Communication System—PECS); dealing with the sensory needs and deviances (e.g., Sensory Integration Therapy and Diet, relevant environmental accommodations, etc.) and increasing the predictability to reduce anxiety (e.g., with the use of visual schedules, calendars and other visual cues). It is fundamental towards this preventive goal to identify at an early stage and deal with common medical and psychiatric comorbidities and conditions, such as general health and pains, seizures, constipation and other gastrointestinal (GI) symptoms, sleep problems, ADHD, intellectual disability, learning disorders, bullying, anxiety, etc. [99,100].

(3) Facilitate and preserve parental acceptance and engagement in the support of their children. Parental engagement is an indispensable component both for parent mediating programs and for professional-based ones, for generalising the acquired skills and for reducing the financial and time encumbrances. However, parental training and psychoeducation should not be delivered in a vacuum. Reduced adaptive adjustment—acceptance and increased feelings of blame and despair—critically influence both the psychological (anxiety, depression, etc.) and somatic wellbeing of the parent [101], impairing the parent’s ability to participate effectively in the child’s management and training. Thus, professional’s training “demands” to parents should be always accompanied by measures aiming at increasing acceptance and ensuring the wellbeing of the parents [97].

(4) Attain a level of academic learning that is commensurate with the individual’s actual cognitive abilities. Although ASD is famously impersonated by people with savant skills and individuals with exceptional academic achievements, the reality is that there is a considerable heterogeneity in their academic achievements, with a substantial danger of underperforming [102]. The covering of the previously mentioned goals to obtain appropriate abilities and behaviours should be coupled with appropriate support (e.g., visual aids, extra academic or psychological sessions, prevention of bullying, social skills training at school [103], etc.) and relevant educational and environmental accommodations to secure a successful inclusion in the appropriate for each child school setting [97].

#### 2.3.2. Older Children and Adolescents

In this developmental period, most of the previous goals continue to be pursued, but the interventions should be directed more towards specific solutions for the rising problems in the here and now. It is imperative that there be a continuation of care for the consistency of which measures should be taken. As the individual grows, there is a big diversity in the overall clinical picture, with some having more problems but others doing as well or even better [104].

Towards the end of primary school, there may be a tendency to reduce or stop specific interventions, especially as the family gets tired or the child is sometimes less willing to attend. However, this may have major repercussions, since light stresses (e.g., changing school setting) may have disproportional effects like catatonia [105]. Thus, parents and professionals should be more cautious and meticulous in preparing individuals for changes to prevent behavioural relapses. Training and emotional support of the parents (in groups or individually) should continue in this phase. The needs of their growing children are changing (e.g., emergence of sexuality), and their own emotional needs continue to impact them and their relationships. Furthermore, the diagnosis and its implications should be reasserted frequently, as parents often tend to “forget” this based also on their assumption of “maturation by age” or that they can grow out of it. According to a recent study, less than 0.5% of children lose the diagnosis of autism, with 6.5% more just losing it to another neurodevelopmental disorder [106].

However, especially in higher-functioning individuals, more complex needs also emerge that can endanger the goals mentioned for the previous period (autonomy, avoidance of challenging behaviours and academic achievements). These needs comprise the renegotiation of all established relationships (a common need for adolescents), body changes, sexuality and more complex peer relationships. There is no doubt about the prevention value of preparing the preadolescent individual with autism (and his/her family) for the upcoming body changes, the self-care and hygiene needed and the new rules that must be followed to stay safe [107]. The same holds true for sexuality education, preventive measures for sexual abuse and recognising early the signs of it [108], as well as exploring sexual orientation and identity. There have been reports of a higher relevant diversity among people with ASD compared to their neurotypical counterparts [109]. 

During this period, and for a substantial part of high-functioning ASD individuals, although a longing for a social life and relationships is present, social and relationship-based difficulties tend to increase, leading to a deeper sense of isolation and frustration thereafter [110]. Social skills training (e.g., Program for the Education and Enrichment of Relationship Skills -PEERS [111]) can prevent further isolation of the individual and protect his/her future quality of life. Pertinent to the above are the related issues of self-esteem and the knowledge and acceptance of their own diagnosis, both of which should be clear preventive targets through psychotherapy and psychoeducation. People with ASD have lower self-esteem, endangering them further to other psychopathological manifestations (e.g., depression, anxiety, etc.) [112]. On the other hand, knowing and coming to terms with their own diagnosis is not only a good practice but is often an imperative need for teenagers with high-functioning autism. The use of an appropriate strategy to do so can enhance ASD self-awareness without impairing self-esteem [113] and prevent chronic frustration over a series of misconceptions and wrong assumptions for their difficulties.

A final preventive target for older children and adolescents is the meticulous screening for any comorbid psychopathology emerging during these rapidly changing years. Additional to the diagnoses reported to concur in the previous stage, we can have disruptive, impulse control and conduct disorders; obsessive-compulsive disorder; bipolar disorders and schizophrenia spectrum disorders, while depression and anxiety are more prevalent than before [99]. Given the difficulty of recognising these often-overshadowed additional symptoms, especially in lower-functioning individuals, a diagnosis can be missed with detrimental effects on the overall functioning of the person. If, however, the comorbidities are noticed, then suitable interventions should be put into place, comprising—apart from psychopharmacology—cognitive-behavioural therapy (CBT) and mindfulness-based interventions [114,115].

#### 2.3.3. Transition to Adulthood

The required additional interventions for this critical period aim to prevent the CDC-described dismal situation for young autistic adults: high rates of unemployment or underemployment and low participation in education beyond high school, while the majority continue to live with family members. This is particularly pertinent to those with higher cognitive ability, who, although they are those who need the above, are often deprived of special support from an adult healthcare system that lacks relevant knowledge and funding [97,116].

For the lower-functioning individuals, a clear plan should be drawn comprising educational and vocational parameters and living circumstances, factoring in their cognitive abilities and adaptive skills, their likes and dislikes, the parents’ realistic expectations and the available resources in the community. This plan can presuppose prevocational and vocational training, followed by a range of options from competitive employment (with some support) to work in sheltered workshops or day services and from semi-independent living (with support) to a totally supervised living environment. In the case of work in the community, they may need help in securing and training on the actual job, as well as liaising with employers and other employees and personal ongoing support, to prevent adverse events that can lead to a loss of the job (e.g., the TEACCH program or Project SEARCH) [117].

Things are more complicated for the individuals at the higher end of the spectrum, and measures should be introduced along the triptych education, employment and independent living. A first step should be a career counselling assessment by professionals with sound knowledge both in counselling and the disorder, using standardised assessment tools—specifically, designed tools for ASD like the Autism Work Skills Questionnaire AWSQ [118]—and qualitative methods to overcome social communication difficulties, so as to facilitate person–job matching [119].

The next step, depending on the career decision reached by the individual (and, to some extent, in collaboration with his family) could either lead to further education on the relevant level (postsecondary vocational training, college, university, etc.) or directly to work placement. For the first part, the successful completion of further schooling, both academically and socially, needs supportive measures directed both at the students (e.g., social planning and organisation skills [120]) and to the academic environment [121]. The level and quality of such support offered in few colleges and universities could underpin the difference between a successful and socially active autistic student and a dropout and/or someone frustrated and depressed [97].

The part of finding and keeping a job (especially a skilled and normally paid one) can be very challenging for people with ASD, however high their cognitive abilities may be. The level of support offered to prevent unemployment varies, depending not only on the actual needs of the individual but, also, on the available resources of the community. It is worth noting that such support, as well as “reasonable accommodations” to employees with disabilities, i.e., without imposing a disproportionate burden to the organisation, are an obligation under Article 5 of the United Nations Convention on the Rights of Persons with Disabilities and are incorporated in the relevant USA law and EU directive. Towards this end, community coaching and employment assistance interventions are offered like the ones mentioned above [117], while interview trainings are opted for to overcome a major burden in getting a job [122]. A more comprehensive approach is supported employment schemes with individualised job placements, prior job training and advocacy and long-term support to ensure job retention [123]. These more intensive programs have good long-term effects on employment and, thus, were found to be cost-effective [124].

The final challenge (correlating with the previous ones) for a successful transitional plan is independent living. Apart from the physical housing problem, independent living for people with ASD comprises a series of other important relevant issues like tasks of day-to-day living, socialising, access to health and welfare services and dealing with the criminal justice system [116]. There is a need for a systematic and thorough assessment of the overall adaptive skills of the person who is about to move out from his parental home, as well as of the available community resources so as to determine the type of independent living he/she will adapt to, i.e., type and location of residential arrangements, commuting needs, funding schemes, type and amount of support needed, therapeutic supporting team, etc. [125]. The final plan must be put together with the utmost participation of the autistic person him/herself.

The above triptych represents the basic challenges in the transition to adult life. Its successful settlement, along with that of the previously mentioned continuous needs (social skills, comorbidities vigilance and treatment and environmental accommodations), are the only ways for a smooth transition to a successful and happy life.

### 2.4. Adulthood and Old Age

The main prevention target in this last period (the longest of all) is the achievement of a good quality of life (QoL). Even after a successful transition to adulthood, pressing needs remain, as recently stated in relevant laws both in the USA (Autism CARES Act) and in the UK (Autism Act). Based on these laws, the community is urged to take supportive measures for adults with ASD without intellectual impairment, so as to expand and retain their autonomy and participation and, also, to prevent mental and physical health deterioration [116]. 

#### 2.4.1. Prevention for Adult Ages Consists of

(1) The continuation of the practical measures described for the transition period (housing, employment and social and everyday functioning) through relative initiatives by the local community and support by the welfare system. Meaningful employment can be key in improving the quality of life, offering structure, social inclusion and participation, financial stability and independence, upgraded housing and leisure activities, better subjective well-being and the protection of their psychological and physical health [97,126]. 

(2) The enhancement of the social skills attained in the previous stages and their adaptation to the new age context. Social skills group interventions seem to be effective at increasing social skills knowledge, as well as social participation [127]. 

(3) Comorbidities vigilance and early treatment. This is also fundamental in this stage, as the prevalence of both physical and mental health conditions in adult life is high, regardless of the presence of intellectual disability [128]. Persons with ASD present risk factors inherent to their diagnosis (deficit in the expression of feelings and thoughts, rigidity, etc.) that, along with relevant environmental stressors (demands to “fit in”, “camouflaging”, bullying, social exclusion, etc.), lead to more psychiatric symptoms, especially depression and anxiety. These can be mitigated with preventive measures, such as programs that increase social participation and leisure activities [129], or psychotherapeutic interventions (adapted CBT and mindfulness) [97] before using medications. It is noteworthy that individuals with high-functioning ASD (especially women) report suicidal ideations and plans at higher rates than the normal population or psychotics, and this is even in the absence of depression [130]. Moreover, suicide is one of the leading causes for higher mortality rates in the ASD population (2.5-fold increase compared to the general population), along with the elevated prevalence of general health problems from almost all systems, with neurological epilepsy being the most common [131]. The increased prevalence of physical diseases not only leads to higher mortality (16 years lost) but, also, impacts the degradation of their QoL. The extent to which these higher morbidities and mortalities reflect an insufficient awareness and diagnosis from health providers and decreased help sought from the autistic population (communication difficulties and access opportunities), as well as health and lifestyle ASD-related issues, show that they can be clear targets for prevention.

#### 2.4.2. Data for Autism in Old Age is Scarce

While there is ambiguity on the trajectories of the neuropsychological indices of autism, there may be an improvement in some symptoms, such as restricted and repetitive behaviours and sensory abnormalities, probably through compensation [132]. However, the accumulated problems described for the previous stages (economic, social, affective and environmental and physical and mental health, plus the effects of the long-term use of antipsychotics) and the characteristics of the disorder, paired with the diminishing until disappearance of supportive interventions, exacerbate the needs and risks pertinent to neurotypical old age. Thus, in this age phase, people with autism are at a higher risk for exclusion and isolation, more psychiatric symptoms (contrary to what is expected with age), less well-managed physical adversities and a sudden realisation of their body changes that may terrify them. Most of the measures proposed for all aging people (healthy lifestyle, balanced diet, regular exercise, participation in social activities, attendance to any medical needs) should also be implemented in the ASD population. Furthermore, we should be vigilant of the possible deterioration in hearing or vision and readily compensate for them. A relevant supportive group can also facilitate the autistic senior to come to terms with his changing situation.

A good example of how successful preventive measures throughout the lifespan can preserve a good QoL for ASD populations can be found in the 2010 article in Atlantic magazine about Case No1 in the original 1943 paper by L. Kanner, Donald T, then aged 77 [133]. Donald was what today we would describe as medium-functioning, with a terse, often repetitive language, a high interest in numbers with savant math skills, an excellent rote memory and favouring spinning objects or himself, who was initially institutionalised for one year. He then passed four years on a rural farm where his caregivers used his preoccupations constructively to do useful things. Both there and later in his own small town, he attended school, where his peculiarities were accepted without bullying. He went on to finish college and work as a teller (again using his fascination for numbers) in his family bank. Donald, at the time of article publishing, was living alone with financial support from a special fund set by the family, had daily morning coffee with friends with minimal or no verbal exchanges, drove daily to play golf and was also one of the most travelled Americans (setting up to six days to visit places and take his own photos of things he saw in pictures). Although Donald’s success story draws heavily from the determination and the wealth of his parents (which can be substituted, in most cases, by the community welfare system), it was his parents’, fellow students’ and fellow townsmen’s acceptance and chances for social participation that helped Donald to continue developing and preserve an outstanding quality of life.

Although more adults and seniors have the diagnosis of autism, and given that adulthood is the longest period of life, making it the most resource demanding, it is the period of life that is scarcely researched and with the fewest allocated resources. Comprehensive interventions aimed at an optimal QoL should be designed and delivered, addressing all the principal components of human life [97], as detailed above. Tertiary prevention for this population is crucial for the wellbeing of the individual, and in most cases, it has proven to be a cost-effective approach.

## 3. Conclusions

The present paper opted to summarise the preventive opportunities for ASD within the lifespan of an individual. There is evidence that primary prevention from the preconception period until the perinatal one can decrease the incidence of autism. However, given the intense variability in the quality of data published and the often-contradictory results presented, we propose the formation of a panel of experts with an ongoing assessment of the published data on the risk factors for ASD and the production of relevant state-of-the-art preventive guidelines for each one of them. Apart from the obvious gains of such an endeavour, it will also facilitate the carrying out of more accurate prevention intervention trials.

Specific very early interventions (<three years of age) targeting the underlying mechanisms of the full phenotype have shown promise as a secondary prevention to reduce ASD prevalence by returning their development to more normalised trajectories and not merely teaching non-fully functioning compensatory skills. The main issue in this approach lies not only on the development and the availability of such early interventions but, also, in early identification—if not of ascertained cases, then at least of those at risk—so the onset of intervention will not be delayed.

We argue that all interventions throughout the lifespan have an inherent tertiary prevention quality against the goals of which their effectiveness should be measured. A key component in this conceptualisation is the increase of adaptive functioning (better behavioural and psychosocial functioning) through a relevant compensation in skills, behaviours and impairments, as well as through an increase in understanding and acceptance from their environment (parents, school, peers and community). In order to be productive for and protective of the QoL of the person with autism, these interventions should be continuous (not stopped upon entering adult life), comprehensive and coordinated; based on the developmental realities and needs of each phase; supported by the welfare system and coupled with a constant vigilance for medical or psychiatric comorbidities or complications. As Lai et al. (2020) pointed out, these interventions should aim at “maximizing potential, minimizing barriers, and optimizing the person-environment fit” [93]. The paths of these endeavours, though laborious, will only result in the betterment of the QoL of a person with autism and will give them the opportunity to exist in society on par with other individuals with dignity.

As research data and clinical experiences accumulate, prevention options are shown to be not only attractive but also a realistic option for such a heterogeneous and dilapidating condition, especially in the absence of an aetiological therapy. Thus, prevention strategies should be a priority for researchers, advocates and stakeholders, as well as public health. 

## Data Availability

Not applicable.
